# Preface to the Special Issue

**DOI:** 10.4103/0971-6203.54841

**Published:** 2009

**Authors:** A S Pradhan

**Affiliations:** Editor-in-Chief, JMP

This special issue of Journal of Medical Physics (JMP) includes manuscripts of some of the presentations at the International Conference on Medical Physics (ICMP-2008) and 29^th^ Annual Conference of Association of Medical Physicists of India (AMPI) entitled *Advanced Technology of Radiation Medicine and Medical Physics Practices*, held during November 26-29, 2008 at Mumbai, India.

Ever since the first meet at Calcutta in December (10 to 12), 1977, annual AMPI conferences have been a big scientific festivity for medical physicists in India. Some of the annual conferences of AMPI occasionally became regional or international conferences depending on the availability of resources and topical need for enhancement of interaction with medical physicists from other countries. International conferences have become more frequent because of rapid development in this field needing larger interaction among professionals (scientists, engineers, suppliers of equipments, oncologists and physicists). Despite the surge in conferences held on topics related to Medical Physics, in India, full text of the papers presented at these conferences have rarely been brought out. The usual practice in these events is to bring out only abstract / synopsis book at the time of the conference.

The organizers of ICMP-2008 decided to bring out papers presented at the conference, in full, as a special issue of the journal (JMP). Publication of full papers based on presentations at conferences/workshops has multiple advantages. For the authors, the quality of work may considerably improve through the seriousness of discussions with peers during the conference. For referees, it becomes easier to review the papers as referees are generally drawn from among the experts attending the conference. To provide equal opportunity to all authors of the invited and proffered papers of ICMP-2008, an announcement on the special issue was included in the conference circulars right from the start. Publishing was optional and the decision was left to authors. However, from among 37 invited papers and 157 proffered papers planned for presentation at the conference (attended by 650 delegates), only a few authors chose to submit their manuscripts for publication in JMP. From the 20 manuscripts received by the amended date, 16 completed the review process and met the norms and the rigor of publication formalities. In this regard, special appreciation is due to invited speakers from abroad (who largely contribute to this special issue) for patiently revising and re revising manuscripts to meet the comments / suggestions of the referees and responding to meet all the requirements of the journal.

The topics covered in this special issue include: Advances in radiation therapy dosimetry, Standardization of radiation based bio-imaging, Advances in PET instrumentation and multi-modality imaging systems, Dose optimization in IMRT, Co-60 tomotherapy – development and application, Experience with Leksell Gamma Knife Perfexion, Tomotherapy for breast irradiation, Commissioning experience and quality assurance of helical tomotherapy machines, Standardization of 2D ion chamber matrix (I'mRT Matrix) for IMRT verification, Radiation safety aspects of PET scanner, Use of iPOD and flash drive for storage of medical images, A novel method for determination of attenuation coefficient, Studies on indigenous production of high specific activity Co-60 teletherapy sources, Measurement of bone density using gamma ray scattering and QA of Tomotherapy HI-ART II helical tomotherapy system. It is hoped that this JMP issue will make a good reference material and be of great use for medical physicists.

